# Trends and outcomes of postpartum haemorrhage, 2003-2011

**DOI:** 10.1186/s12884-015-0788-5

**Published:** 2015-12-15

**Authors:** Jane B. Ford, Jillian A. Patterson, Sean K. M. Seeho, Christine L. Roberts

**Affiliations:** University Department of Obstetrics and Gynaecology, Royal North Shore Hospital, Building 52, St Leonards, NSW 2065 Australia

**Keywords:** Maternal morbidity, Postpartum haemorrhage, Transfusion

## Abstract

**Background:**

While rates of postpartum haemorrhage (PPH) have continued to rise, it is not clear if the association with other morbidity and transfusion has changed over time. This study explores the recent trend in postpartum haemorrhage and whether postpartum haemorrhage is associated with increased transfusions or adverse outcomes over time.

**Methods:**

Linked birth and hospital data were used to examine ICD-10 AM coded PPH and outcomes in maternal birth admission records, 2003-–2011 in hospitals in New South Wales (NSW), Australia (*N* = 818,965 pregnancies). Trends were calculated on the whole population, and among subgroups, and tested using the Cochran Armitage test for trend. Logistic regression models were developed separately for vaginal and caesarean births, and for a maternal morbidity composite indicator (excluding transfusion) and red cell transfusion. Adjusted odds ratios (aOR) for each year relative to 2003 and 95 % confidence intervals (CI) are presented with adjustment for maternal (eg. age, country of birth) and pregnancy factors (eg. parity, interventions, pregnancy complications).

**Results:**

Overall, there was a significant increase in the PPH rate, from 6.1 % in 2003 to 8.3 % in 2011 (*p* < 0.0001). Crude rates of postpartum haemorrhage with transfusion increased from 0.75 % (*n* = 636) to 1.21 % (*n* = 1145) (*p* < 0.0001) while crude rates of postpartum haemorrhage with maternal morbidity increased from 0.18 % (*n* = 149) to 0.23 % (*n* = 221) (*p* = 0.02). Having accounted for maternal and pregnancy factors, there were significant overall decreases in the odds of *morbidity* among women with a PPH delivering vaginally (in 2006, 2007 and 2010, aORs were 0.70 (95 % CI 0.52, 0.96) 0.69 (0.51, 0.94) and 0.64 (0.47, 0.87) relative to 2003; *p* < 0.05), and no significant decrease among women delivered by caesarean section (aOR 0.87 (0.58, 1.29) in 2011; *p* = 0.37). Among women with a PPH delivering vaginally, there was a trend towards a non-linear increase in the adjusted odds of transfusion by birth year. Compared to women who had vaginal births with PPH in 2003, the adjusted odds for transfusion was between 1.1 and 1.2 fold higher for those with a PPH delivering vaginally in 2007, 2009, 2010 and 2011. However there was no significant trend amongst caesarean births (aOR 0.84 (0.66, 1.06) in 2011; *p* = 0.29).

**Conclusions:**

PPH has become more frequent, however this has not been associated with a clear pattern of increased severe maternal morbidity. This suggests that the increase in PPH may represent fewer severe haemorrhages, better management of severe haemorrhage or better recording of PPH. The increase in transfusions following vaginal births with PPH warrants further investigation.

**Electronic supplementary material:**

The online version of this article (doi:10.1186/s12884-015-0788-5) contains supplementary material, which is available to authorized users.

## Background

Postpartum haemorrhage rates have increased in the developed world [1]. At the same time, transfusion rates associated with childbirth have risen in Australia [2], Canada [3] and the United States [4]. It is unclear whether the increased rates of postpartum haemorrhage represent more severe haemorrhage. In Australia, the postpartum haemorrhage rate increased by around 25 % from 1994 to 2002. During this period, the transfusion rate among women with postpartum haemorrhage increased by 500 % (from 2 to 12 %) [5]. Obstetric transfusions (not limited to women with postpartum haemorrhage) increased from 1.2 % of births in 2001 to 1.6 % of births in 2010 [2].

Current population-based pregnancy and birth data do not include details of the amount of blood lost at postpartum haemorrhage, nor the quantity of blood transfused. In the absence of these details, post-haemorrhage outcomes such as maternal morbidity and transfusion may provide insight into whether bleeding is becoming more severe over time. Given increasing caesarean section rates and different risk factors reportedly associated with vaginal and caesarean births [6, 7], it is worthwhile considering the outcomes following postpartum haemorrhage separately for these birth modes.

The aims of this study were to determine the recent trend in postpartum haemorrhage and whether postpartum haemorrhage is associated with increased transfusions or severe adverse outcomes over time.

## Methods

The study population consisted of all women with livebirths or stillborn infants of at least 20 weeks gestation in New South Wales (NSW) hospitals between 2003 and 2011. Data on maternal demographics, the pregnancy, labour and birth were obtained from the Perinatal Data Collection (‘Birth data’). This is a legislated statewide record of all births in NSW, and is completed by the midwife or doctor at the time of birth. Data on maternal diagnoses and procedures were obtained from the Admitted Patients Data Collection (‘Hospital data’), which contains data collected on all private and public hospital discharges in NSW. Diagnoses and procedures associated with hospital discharges are coded according to the 10^th^ revision of the International Classification of Diseases, Australian Modification (ICD10-AM) [8] and the Australian Classification of Health Interventions (ACHI) [9] respectively.

The Centre for Health Record Linkage performed probabilistic linkage of the hospital and birth data, to assign unique project identifiers to each mother, allowing both longitudinal linkage of pregnancies and hospitalisations during and following pregnancy. For this study, rates of missed and incorrect links were less than 0.5 %.

Postpartum haemorrhage was identified from the hospital data using ICD codes (O72 and subcodes). Blood loss of equal or greater than 500 mL following a vaginal birth, or equal or greater than 750 mL following a caesarean birth is classified as a postpartum haemorrhage [10]. Validation studies have indicated that postpartum haemorrhage is accurately reported, although under-enumerated with sensitivity and specificity of 73.8 % and 98.9 %, respectively [11]. Previous postpartum haemorrhage was defined as a postpartum haemorrhage reported in any previous pregnancy (since July 2001) and was obtained from the longitudinal linkage of a woman’s pregnancies.

Outcomes of interest were severe maternal morbidity and blood transfusion. Maternal morbidity was measured using a validated composite indicator of adverse outcomes [12]. This indicator includes the following diagnoses: acute abdomen, acute renal failure, acute pyschosis, cardiac arrest, cerebral oedema or coma, disseminated intravascular coagulopathy, cerebro-vascular accident, major complications of anaesthesia, obstetric embolism, shock, status asthmaticus or epilepticus or uterine rupture; as well as procedures including: assisted ventilation, dialysis, evacuation of haematoma, hysterectomy, dilatation and curettage (with general anaesthetic), procedures to reduce blood flow to uterus, reclosure of disrupted CS wound, repair of bladder or cytostomy, repair of intestine or repair of ruptured or inverted uterus. The original indicator included blood transfusion as a component of morbidity, however this was excluded from the criteria for morbidity and examined separately for this study. Use of a composite indicator overcomes the under-ascertainment of individual diagnoses and procedures [13]. Blood transfusion was defined in this study as transfusion of whole blood or red blood cells (ACHI 13706-01, 13706-02) [9], which is well reported (sensitivity and specificity of 83.1 % and 99.9 %, respectively) [11]. Since we were interested in trends in transfusion and morbidity, women experiencing both a transfusion and a morbidity diagnosis were included in both the transfusion and morbidity outcomes. A sensitivity analysis was performed to assess whether estimates changed when transfusion and morbidity were treated as mutually exclusive outcomes.

Maternal demographics, pregnancy, labour and delivery data were obtained from the birth data; chronic conditions (chronic hypertension, diabetes, renal and cardiac conditions), antepartum haemorrhage (APH), morbidly adherent placenta, and placenta praevia were obtained from the hospital data; and pregnancy hypertension and gestational diabetes were obtained from either birth or hospital data based on validation studies indicating the most reliably reported sources [11, 14–17]. Pregnancy, labour and delivery data have been shown to be well reported in these datasets [18, 19].

Trends were calculated on the whole population (including multifetal pregnancies), and among subgroups, and tested using the Cochran Armitage test for trend. Logistic regression models were developed to assess the change in severity of postpartum haemorrhage over time (measured by maternal morbidity or transfusion) among birth admissions for women delivering a singleton infant and experiencing a postpartum haemorrhage. Modelling was restricted to singleton births given the different risk profile of women with multifetal pregnancies. Models were developed separately for vaginal and caesarean births given the different blood loss criteria for postpartum haemorrhage diagnosis in Australian data (≥500 mls post-vaginal birth, ≥750 mls post-caesarean). Separate models were also developed for maternal morbidity and transfusion. All available risk factors for morbidity/ transfusion were entered into the multivariable models. A sensitivity analysis was also performed including diagnosis codes for placenta praevia with haemorrhage as part of the definition of a postpartum haemorrhage, however associations were unchanged so this analysis has not been included. All analysis was conducted in SAS 9.3. Ethics approval was from the NSW Population and Health Services Research Ethics Committee who deemed that individual consent was not required for this study (#2006-06-011).

## Results

**Fig. 1:**
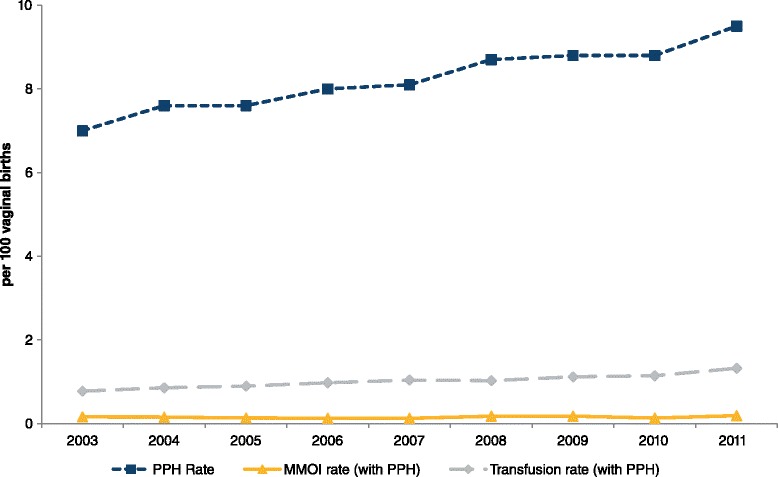
Trends in postpartum haemorrhage, adverse maternal outcomes (MMOI) and red cell transfusion rates among vaginal births in NSW, 2003–2011. MMOI – Maternal morbidity outcome indicator

**Fig. 2:**
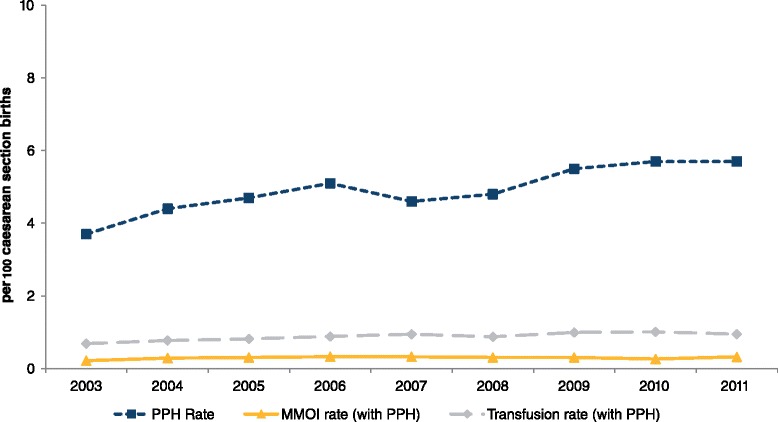
Trends in postpartum haemorrhage, adverse maternal outcomes (MMOI) and red cell transfusion rates among caesarean section births in NSW, 2003–2011. MMOI – Maternal morbidity outcome indicator

Between 2003 and 2011 there were 818,965 births in New South Wales. Overall, a postpartum haemorrhage was reported in 59,639 (7.3 %) of births. There was a significant increase in the postpartum haemorrhage rate, from 6.1 % (*n* = 5158) in 2003 to 8.3 % (*n* = 7886) in 2011 (*p* < 0.0001). Rates of postpartum haemorrhage with transfusion increased from 0.75 % (*n* = 636) to 1.21 % (*n* = 1145) (*p* < 0.0001) while rates of postpartum haemorrhage with maternal morbidity increased from 0.18 % (*n* = 149) to 0.23 % (*n* = 221) (*p* = 0.02). Among caesarean births, the rate of postpartum haemorrhage increased by 55 % (3.7 % to 5.7 %), whereas among vaginal births they increased by 36 % (7.0 % to 9.5 %) (Figs. 1 and 2). Women with a postpartum haemorrhage were more likely to have multifetal pregnancies, be primiparous, have pregnancy hypertension, antepartum haemorrhage, instrumental delivery and large-for-gestational age infants, and were less likely to be a private patient. Women with a previous postpartum haemorrhage were more likely to have a postpartum haemorrhage in the current pregnancy than women without a history of postpartum haemorrhage (Table 1).

**Table 1:** Maternal and pregnancy characteristics of women with and without postpartum haemorrhage (PPH), NSW, 2003–2011

Variable	Postpartum haemorrhage (*N* = 59,639)	No postpartum haemorrhage (*N* = 759,326)
Maternal age (years)		
Under 20	2386 (4.0)	27407 (3.6)
20–34	45012 (75.5)	562067 (74.0)
35+	12241 (20.5)	169852 (22.4)
Smoker	6524 (10.9)	88992 (11.7)
Australian born	38530 (64.6)	523266 (68.9)
Multiple birth	1647 (2.8)	10927 (1.4)
Parity		
1st	29179 (48.9)	317620 (41.8)
2nd-4th	28021 (47.0)	412846 (54.4)
5+	2366 (4.0)	27588 (3.6)
Previous Caesarean	5489 (9.2)	111610 (14.7)
Gestational diabetes	3842 (6.4)	45688 (6.0)
Pregnancy hypertension	6740 (11.3)	69387 (9.1)
Antepartum haemorrhage	2440 (4.1)	19246 (2.5)
Malpresentation	2037 (3.4)	35981 (4.7)
Previous PPH	10823 (18.1)	27116 (3.6)
Placenta praevia	1253 (2.1)	7678 (1.0)
Morbidly adherent placenta	1163 (2.0)	826 (0.1)
Gestational age (weeks)		
20–32	1372 (2.3)	11654 (1.5)
33–36	3023 (5.1)	37969 (5.0)
37+	55244 (92.6)	709703 (93.5)
Mode of birth		
Normal vaginal delivery	36717 (61.6)	451652 (59.5)
Caesarean	11860 (19.9)	228010 (30.0)
Caesarean without labour	5465 (9.2)	133292 (17.6)
Caesarean with labour	6393 (10.7)	94704 (12.5)
Instrumental delivery	11186 (18.8)	80382 (10.6)
Forceps	4802 (8.1)	26272 (3.5)
Vacuum	6384 (10.7)	54110 (7.1)
Induction of labour	20479 (34.3)	202866 (26.7)
Large for gestational age	9197 (15.4)	78804 (10.4)
Hospital type		
Tertiary	31503 (52.8)	307664 (40.5)
Regional	11561 (19.4)	161522 (21.3)
Urban/other	8754 (14.7)	99859 (13.2)
Private	7821 (13.1)	190281 (25.1)

### Subsequent morbidity among women with a postpartum haemorrhage

There was no significant change in the crude maternal morbidity rate (excluding transfusion) among all women with a postpartum haemorrhage (*p* = 0.28) (2.9 per 100 births to 2.8 per 100 births). Having accounted for maternal and pregnancy factors, there were significant decreases in the odds of morbidity among women with a postpartum haemorrhage (PPH) delivering vaginally (in 2006, 2007 and 2010 aORs were 0.70 (0.52, 0.96), 0.69 (0.51, 0.94) and 0.64 (0.47,0.87) relative to 2003; *p* < 0.05), and no significant decrease among women delivering by caesarean section (aOR 0.87 (0.58, 1.29) in 2011 compared to 2003; *p* = 0.37) (Table 2).

**Table 2:** Year of birth and *maternal morbidity* ^a^ among women with a singleton pregnancy and postpartum haemorrhage, NSW, 2003–2011

	Vaginal births *N* = 46955		Caesarean births *N* = 11037	
	Unadjusted OR	Adjusted OR^b^	Unadjusted OR	Adjusted OR^b^
**Year of birth**				
2003	**Ref**	**Ref**	Ref	Ref
2004	**0.87 (0.65, 1.17)**	**0.90 (0.66, 1.21)**	1.20 (0.80, 1.79)	1.13 (0.74, 1.72)
2005	**0.76 (0.56, 1.03)**	**0.79 (0.58, 1.08)**	1.16 (0.78, 1.71)	1.14 (0.76, 1.73)
2006	**0.70 (0.52, 0.94)**	**0.70 (0.52, 0.96)**	1.13 (0.77, 1.65)	1.03 (0.69, 1.55)
2007	**0.70 (0.52, 0.94)**	**0.69 (0.51, 0.94)**	1.30 (0.89, 1.90)	1.10 (0.73, 1.65)
2008	**0.93 (0.70, 1.22)**	**0.90 (0.78, 1.20)**	1.18 (0.81, 1.73)	1.04 (0.69, 1.55)
2009	**0.91 (0.69, 1.20)**	**0.91 (0.69, 1.21)**	1.00 (0.69, 1.46)	0.87 (0.58, 1.30)
2010	**0.65 (0.48, 0.88)**	**0.64 (0.47, 0.87)**	0.78 (0.53, 1.16)	0.67 (0.44, 1.01)
2011	**0.86 (0.65, 1.13)**	**0.86 (0.65, 1.14)**	1.02 (0.70, 1.48)	0.87 (0.58, 1.29)
**Maternal Age**				
Under 20		**1.02 (0.71, 1.46)**		**0.39 (0.14, 1.08)**
20–34		**Ref**		**Ref**
35+		**1.31 (1.10, 1.55)**		**1.16 (0.97, 1.40)**
**Private patient**		0.89 (0.69, 1.14)		0.92 (0.69, 1.21)
**Smoker**		1.02 (0.82, 1.27)		1.12 (0.86, 1.45)
**Parity**				
1				
2–4		0.87 (0.74, 1.03)		**1.29 (1.04, 1.64)**
5+		0.80 (0.55, 1.17)		**1.53 (1.04, 2.24)**
**Australian born**		**0.82 (0.70, 0.96)**		**0.72 (0.60, 0.87)**
**Gestational Age**				
20–32 weeks		**2.43 (1.62, 3.65)**		**2.27 (1.61, 3.22)**
33–36 weeks		**1.28 (0.92, 1.79)**		**1.52 (1.16, 1.98)**
37+ weeks		**Ref**		**Ref**
**Mode of birth**				
Vaginal		Ref		
Forceps		1.19 (0.95, 1.50)		.
Vacuum		1.14 (0.92, 1.41)		.
Caesarean in labour				**1.64 (1.32, 2.03)**
Pre-labour caesarean				
**Induction**		1.10 (0.95, 1.27)		**1.31 (1.03, 1.67)**
**Large for gestational age**		0.84 (0.68, 1.04)		0.82 (0.65, 1.03)
**Chronic conditions**		**2.94 (2.08, 4.14)**		**2.07 (1.45, 2.97)**
**Pregnancy factors**				
Antepartum haemorrhage		**1.42 (1.02, 1.96)**		1.04 (0.78, 1.39)
Previous caesarean		**1.77 (1.33, 2.35)**		**1.80 (1.43, 2.26)**
Previous PPH		**1.48 (1.25, 1.75)**		**1.75 (1.44, 2.12)**
Malpresentation		1.39 (0.59, 3.28)		1.01 (0.78, 1.29)
Morbidly adherent placenta		**4.25 (3.20, 5.64)**		**10.21 (7.78, 13.40)**
Placenta praevia				**2.31 (1.80, 2.96)**
Gestational diabetes		1.12 (0.85, 1.46)		0.94 (0.70, 1.27)
Pregnancy hypertension		**1.52 (1.24, 1.85)**		**1.58 (1.25, 1.98)**
**Hospital**				
Tertiary		Ref		Ref
Urban		1.15 (0.95, 1.41)		1.00 (0.76, 1.32)
Regional		1.16 (0.96, 1.41)		1.23 (0.95, 1.58)
Private		1.05 (0.77, 1.43)		1.29 (0.90, 1.85)

Numbers in bold represent statistically significant adjusted risk or protective factors for maternal morbidity. Chronic conditions included chronic hypertension, pre-existing diabetes, renal disease, cardiac disease

^a^Maternal morbidity includes diagnoses such as shock, uterine rupture, and cerebrovascular accident as well as procedures including assisted ventilation, dialysis, evacuation of haematoma, hysterectomy, dilatation and curettage under general anaesthesia. A full list of diagnoses and procedures is listed in the methods. ^b^Adjusted for all other variables in the column

Morbidity or transfusion was more common among women with postpartum haemorrhage in the context of multiple birth, previous caesarean, previous PPH, placenta praevia and morbidly adherent placenta (Additional file 1: Table S1). Risk factors for maternal morbidity among women with a postpartum haemorrhage were similar for those giving birth vaginally and by caesarean section. These risk factors included advanced maternal age (>35 years), women with chronic conditions, maternal birth outside Australia, preterm birth, placenta praevia, morbidly adherent placenta, pregnancy hypertension, and women with a history of postpartum haemorrhage. Instrumental deliveries were associated with higher morbidity among vaginal births (Table 2).

### Transfusion among women with a postpartum haemorrhage

There was a significant increase in the red cell transfusion rate (*p* < 0.001) among all women with a postpartum haemorrhage from 12.3 per 100 births in 2003 to 14.5 per 100 births in 2011. Among women with a PPH delivering vaginally, there was a trend towards a non-linear increase in the adjusted odds of transfusion by birth year. Compared to women who had vaginal births with PPH in 2003, the adjusted odds for transfusion was between 1.1 and 1.2 fold higher for those with a PPH delivering vaginally in 2007, 2009, 2010 and 2011There was no significant trend amongst caesarean births with postpartum haemorrhage (aOR 0.84 (0.66, 1.06) in 2011 relative to 2003; *p* = 0.29) (Table 3). Risk factors for blood transfusion following postpartum haemorrhage included the extremes of maternal age, preterm birth, morbidly adherent placenta, placenta praevia, antepartum haemorrhage, induction of labour, previous postpartum haemorrhage, and delivery in a regional hospital. For vaginal deliveries, instrumental births, pregnancy hypertension, smoking and previous caesarean section were also risk factors. For caesarean births, higher parity was associated with increased risk of transfusion (Table 3).

**Table 3:** Year of birth and *red cell blood transfusion* among women with a singleton pregnancy and postpartum haemorrhage, NSW, 2003–2011

	Vaginal births *N* = 46955		Caesarean births *N* = 11037	
	Unadjusted OR	Adjusted OR^a^	Unadjusted OR	Adjusted OR^a^
**Year of birth**				
2003	**Ref**	**Ref**	Ref	Ref
2004	**1.03 (0.90, 1.18)**	**1.03 (0.90, 1.18)**	0.91 (0.70, 1.17)	0.90 (0.67, 1.17)
2005	**1.07 (0.94. 1.22)**	**1.08 (0.95, 1.24)**	0.91 (0.70, 1.16)	0.92 (0.71, 1.18)
2006	**1.13 (0.99, 1.28)**	**1.13 (0.99, 1.29)**	0.92 (0.72, 1.16)	0.88 (0.68, 1.13)
2007	**1.18 (1.04, 1.34)**	**1.15 (1.01, 1.31)**	1.19 (0.94, 1.50)	1.11 (0.87, 1.42)
2008	**1.08 (0.95, 1.22)**	**1.05 (0.92, 1.19)**	1.01 (0.80, 1.26)	0.96 (0.75, 1.23)
2009	**1.18 (1.04, 1.34)**	**1.15 (1.01, 1.31)**	1.01 (0.80, 1.26)	0.97 (0.77, 1.24)
2010	**1.20 (1.06, 1.36)**	**1.16 (1.02, 1.31)**	0.96 (0.77, 1.21)	0.91 (0.72, 1.16)
2011	**1.29 (1.15, 1.46)**	**1.23 (1.08, 1.39)**	0.89 (0.71, 1.11)	0.84 (0.66, 1.06)
**Maternal Age**				
Under 20		**1.29 (1.14, 1.46)**		**1.99 (1.47, 2.69)**
20–34		**Ref**		**Ref**
35+		**1.11 (1.03, 1.20)**		**1.11 (0.99, 1.25)**
**Private patient**		**0.87 (0.78, 0.96)**		0.93 (0.78, 1.10)
**Smoker**		**1.16 (1.07, 1.27)**		0.98 (0.83, 1.16)
**Parity**				
1				
2–4		**0.79 (0.74, 0.85)**		**1.25 (1.09, 1.44)**
5+		**0.84 (0.72, 0.99)**		**2.13 (1.68, 2.71)**
**Australian born**		0.95 (0.89, 1.01)		**0.74 (0.66, 0.83)**
**Gestational Age**				
20–32 weeks		**1.21 (0.95, 1.54)**		**1.79 (1.40, 2.30)**
33–36 weeks		**1.38 (1.20, 1.59)**		**1.07 (0.88, 1.29)**
37+ weeks				
**Mode of birth**				
Vaginal				
Forceps		1.70 (1.55, 1.86)		
Vacuum		1.32 (1.21, 1.43)		
Caesarean in labour				**1.57 (1.37, 1.79)**
Pre-labour caesarean				
**Induction**		**1.12 (1.06, 1.19)**		1.01 (0.87, 1.16)
**Large for gestational age**		**1.09 (1.01, 1.18)**		1.13 (0.99, 1.28)
**Chronic conditions**		**1.27 (1.03, 1.57)**		1.23 (0.94, 1.62)
**Pregnancy factors**				
Antepartum haemorrhage		**1.49 (1.30, 1.72)**		**1.47 (1.23, 1.76)**
Previous caesarean		**1.43 (1.25, 1.63)**		1.03 (0.89, 1.19)
Previous PPH		**1.40 (1.31, 1.51)**		**1.27 (1.12, 1.44)**
Malpresentation		1.07 (0.68, 1.69)		1.03 (0.87, 1.20)
Morbidly adherent placenta		**3.88 (3.32, 4.53)**		**5.21 (4.06, 6.68)**
Placenta praevia				3.09 (2.62, 3.65)
Gestational diabetes		1.00 (0.89, 1.13)		0.92 (0.76, 1.10)
Pregnancy hypertension		**1.38 (1.26, 1.50)**		1.12 (0.97, 1.31)
**Hospital**				
Tertiary		**Ref**		**Ref**
Urban		**1.05 (0.97, 1.14)**		**1.03 (0.87, 1.21)**
Regional		**1.29 (1.20, 1.39)**		**1.54 (1.33, 1.79)**
Private		**0.88 (0.77, 1.00)**		**1.64 (1.31, 2.05)**

Numbers in bold represent statistically significant adjusted risk or protective factors for transfusion. Chronic conditions included chronic hypertension, pre-existing diabetes, renal disease, cardiac disease. ^a^Adjusted for all other variables in the column

A sensitivity analysis (data not shown) was performed in which women experiencing both a transfusion and morbidity (*n* = 925) were only included in the morbidity outcome. The increase in blood transfusion persisted but was slightly attenuated (10.7 to 12.7 per 100 births; *p* < 0.001). The adjusted morbidity and transfusion trends were unchanged.

## Discussion

This population-based study found not only had postpartum haemorrhage rates increased between 2003 and 2011, but that the increase over this 9 year period was proportionally higher than previously reported [20] (36 % increase compared to 25 % in the previous 8 year period). Although the largest number of postpartum haemorrhages occurred among vaginal births in our setting, the increase in the postpartum haemorrhage rate was primarily driven by haemorrhage post-caesarean birth. We found that while the postpartum haemorrhage rate has continued to rise, among women experiencing a haemorrhage post-vaginal birth there has been a decrease in maternal morbidity. However, transfusions among women having a postpartum haemorrhage following vaginal birth increased over the study period.

Despite known under-ascertainment of postpartum haemorrhage for women delivered by caesarean section [21], there are no known reporting changes over the study period. Other studies reporting increases in overall postpartum haemorrhage have not stratified by mode of delivery so it is unknown whether this trend is mirrored in other settings [1, 22]. A recent Canadian paper suggested that post-caesarean atonic haemorrhage increased by 95 % from 2001 to 2009 while post-vaginal delivery atonic haemorrhage increased by 35 % [3]. We have previously demonstrated misclassification of reporting on PPH cause using ICD reporting, in particular over-estimation of the contribution of uterine atony to PPH [23]. There are no known practice changes associated with caesarean delivery over this period that explain increased haemorrhage rates.

Reassuringly, morbidity rates post-haemorrhage do not appear to have increased, and among vaginal deliveries decreased over the study period. This suggests that the increase in postpartum haemorrhage does not represent an increase in severe haemorrhage resulting in life-threatening complications. While more postpartum haemorrhages have been identified, the management of these may be preventing deterioration into severe morbidity. Population data do not include detailed information on staffing or timing of interventions that may have contributed to decreasing maternal morbidity over the period. Previously, postpartum haemorrhage was reported to be driving up rates of maternal morbidity in Australia [13]; importantly, this previous study included transfusion as part of the composite measure of maternal morbidity, whereas for the current study we have investigated transfusion separately.

After adjustment for other risk factors, there was a significant increase in red cell transfusions for vaginal births, however there was no significant trend among caesarean births. Given that the increase among vaginal births occurred in the context of no increase in morbidity, this may reflect an increased frequency of use of red cell transfusion for less severe cases of haemorrhage post-vaginal delivery. It is not clear why an increase in transfusions only occurred following postpartum haemorrhage at vaginal birth (not following caesareans); this may be related to different staff making decisions about transfusions in the labour ward versus theatre, differential use of point of care haemoglobin testing and/or timing of access to additional blood loss minimization techniques such as Bakri balloons.

The proportion of women with postpartum haemorrhage receiving transfusion had previously increased dramatically (from 2 % in 1994 to 12 % in 2002) [5]. Our study suggests that a higher proportion of women are now transfused (12 % in 2003 to 15 % in 2011), however, the rate of increase is much less than previously demonstrated. Without details on actual blood loss or timing of transfusion (immediately post-delivery or a few days postpartum) it is difficult to speculate about the severity of haemorrhages being transfused. Nevertheless, in the setting of increased obstetric transfusions [2] and the largely unpredictable nature of postpartum haemorrhage, patient blood management strategies including recognizing and treating antepartum anemia, active management of the third stage of labour and use of iron therapy in the setting of postpartum anemia are worthwhile considerations for pregnant and recently pregnant women.

While preterm birth and the presence of chronic conditions increased the risk of morbidity following postpartum haemorrhage, even when these factors were taken into account, women with a previous caesarean birth or previous birth complicated by postpartum haemorrhage remained at increased risk of subsequent morbidity. The role previous birth complications play in index birth complications is increasingly being recognised [24, 25] and underlines the importance of recording and taking into account obstetric history when caring for multiparous women.

Strengths of this study include the use of validated population-based data to investigate rare outcomes including severe adverse maternal outcomes and transfusion as well as the incorporation of previous obstetric history. Using record linkage we have been able to take into account previous postpartum haemorrhage and previous caesarean, although for the earlier years of the study only a two year lookback period was available. The two outcomes considered in this study may be inter-related –a transfusion in some cases is likely to have prevented further morbidity. Nevertheless, there are known limitations to using administrative datasets including under-enumeration of haemorrhage, transfusion and other adverse outcomes as well as difficulties in establishing temporality of events. While the amount of blood loss is likely under-estimated [26], more severe haemorrhage is more likely to be reported [11]. Based on previously demonstrated differential under-enumeration of postpartum haemorrhage by mode of delivery [21], we examined outcomes separately following vaginal and caesarean births. However, incidence rates of postpartum haemorrhage, particularly following caesarean birth, may be under-enumerated.

## Conclusions

While women giving birth today are more likely to have a postpartum haemorrhage, on the whole they are no more likely to experience severe adverse outcomes subsequent to the haemorrhage than in 2003. The increase in post-caesarean haemorrhage highlights the need for vigilance in the operative setting. The increase in red cell transfusions following vaginal birth with postpartum haemorrhage warrants further investigation. The increase in PPH with a decrease in severe maternal morbidity (particularly among vaginal births) may represent fewer severe haemorrhages, better management of severe haemorrhage or better recording of PPH. Further insight into the severity of postpartum haemorrhages in the Australian setting will soon be available via record linkage that allows number of units transfused to each woman to be assessed.

## Additional file

Additional file 1: Table S1. Maternal and pregnancy characteristics of women with postpartum haemorrhage (PPH) with and without morbidity/transfusion, NSW, 2003-2011. (DOCX 15 kb)

## Abbreviations

ACHI: Australian classification of health interventions
ICD10-AM: International classification of diseases, Australian modification
MMOI: Maternal morbidity outcome indicator
NSW: New South Wales
PPH: Postpartum haemorrhage
