# Supplementary material for: Trends and outcomes of postpartum haemorrhage, 2003-2011
Source: BMC Pregnancy Childbirth. 2015 Dec 15;15:334. doi: 10.1186/s12884-015-0788-5 (PMC4681164; doi:10.1186/s12884-015-0788-5)
Supplement: Additional file 1: Table S1. — Maternal and pregnancy characteristics of women with postpartum haemorrhage (PPH) with and without morbidity/transfusion, NSW, 2003-2011. (DOCX 15 kb) [file 12884_2015_788_MOESM1_ESM.docx]

**Additional file 1: Table 1. Maternal and pregnancy characteristics of women with postpartum haemorrhage (PPH) with and without morbidity/transfusion, NSW, 2003-2011**

|  | **PPH with morbidity/transfusion** | **PPH without morbidity/transfusion** |
| --- | --- | --- |
| Total | 8686 (100.0) | 50953 (100.0) |
| Maternal age (years) |  |  |
| Under 20 | 428 ( 4.9) | 1958 ( 3.8) |
| 20-34 | 6227 ( 71.7) | 38785 ( 76.1) |
| 35+ | 2031 ( 23.4) | 10210 ( 20.0) |
| Smoker | 1216 ( 14.0) | 6183 ( 12.1) |
| Australian born | 5602 ( 64.5) | 32928 ( 64.6) |
| Multiple birth | 407 ( 4.7) | 1240 ( 2.4) |
| Parity |  |  |
| 1st | 4414 ( 50.8) | 24765 ( 48.6) |
| 2nd-4th | 3810 ( 43.9) | 24211 ( 47.5) |
| 5+ | 447 ( 5.1) | 1919 ( 3.8) |
| Previous caesarean | 1095 ( 12.6) | 4394 ( 8.6) |
| Gestational diabetes | 587 ( 6.8) | 3255 ( 6.4) |
| Pregnancy hypertension | 1300 ( 15.0) | 5440 ( 10.7) |
| Antepartum haemorrhage | 565 ( 6.5) | 1875 ( 3.7) |
| Malpresentation | 435 ( 5.0) | 1602 ( 3.1) |
| Previous PPH | 1998 ( 23.0) | 8825 ( 17.3) |
| Placenta praevia | 498 ( 5.7) | 755 ( 1.5) |
| Morbidly adherent placenta | 528 ( 6.1) | 635 ( 1.2) |
| Gestational age |  |  |
| 20-32 | 379 ( 4.4) | 993 ( 1.9) |
| 33-36 | 657 ( 7.6) | 2366 ( 4.6) |
| 37+ | 7650 ( 88.1) | 47594 ( 93.4) |
| Mode of birth |  |  |
| Normal vaginal delivery | 4425 ( 50.9) | 32292 ( 63.4) |
| Caesarean | 2381 ( 27.4) | 9479 ( 18.6) |
| Caesarean without labour | 1100 ( 12.7) | 4365 ( 8.6) |
| Caesarean with labour | 1279 ( 14.7) | 5114 ( 10.0) |
| Instrumental delivery | 1879 ( 21.6) | 9307 ( 18.3) |
| Forceps | 902 ( 10.4) | 3900 ( 7.7) |
| Vacuum | 977 ( 11.2) | 5407 ( 10.6) |
| Induction | 3091 ( 35.6) | 17388 ( 34.1) |
| Large for gestational age | 1401 ( 16.1) | 7796 ( 15.3) |
| Hospital type |  |  |
| Tertiary | 4587 ( 52.8) | 26916 ( 52.8) |
| Regional | 1917 ( 22.1) | 9644 ( 18.9) |
| Urban/other | 1172 ( 13.5) | 7582 ( 14.9) |
| Private | 1010 ( 11.6) | 6811 ( 13.4) |
